# 1198. Impact of Quaternary Ammonium Disinfectants on Transfer of viruses from Fomites to the Finger

**DOI:** 10.1093/ofid/ofac492.1031

**Published:** 2022-12-15

**Authors:** Charles P Gerba

**Affiliations:** University of Arizona, Tucson, Arizona

## Abstract

**Background:**

Hard and soft surfaces (fomites) can play a role in the transmission of both enteric and respiratory viruses. Transmission can occur by touching of contaminated surfaces and bringing the hand to the either the mouth or nose or by re-aerosolization of the virus from the surface. In adults face touching occurs every 3 to 4 minutes. The amount of virus transfer which occurs depends on the nature of the surface, the virus and the degree and type of activity within a facility.

**Methods:**

We conducted studies on the finger transfers of both a non-enveloped (MS-2 coliphages) and an enveloped virus (coronavirus 229E).

**Results:**

The greatest degree of the non-enveloped virus occurred with acrylic plastic and stainless steel, while the greatest transfer of the enveloped virus occurred from porcelain. Transfer to the finger from surfaces of the coronavirus was reduced by treatment of surfaces with various commercially available quaternary ammonium disinfectants after 24 hours of application to the fomites by 37% to 99.9%. Using MS-2 coliphage as a tracer virus placed on a high touch fomite, we found that the use of quaternary ammonium containing disinfecting wipes in a long term care facility reduced the contamination of fomites by 80%.

**Conclusion:**

The results indicate that spread of viruses in a facility and exposure via hand contact can be significantly reduced by disinfection of fomites.

**Disclosures:**

**Charles P. Gerba, PhD**, Allied Biosciences: Grant/Research Support|Boeing: Grant/Research Support|Corning: Grant/Research Support|GOJO: Grant/Research Support|Rickett: Advisor/Consultant|Rickett: Grant/Research Support.

